# Supplementary material for: Morphological Changes and Component Characterization of Coffee Silverskin
Source: Molecules. 2021 Aug 13;26(16):4914. doi: 10.3390/molecules26164914 (PMC8400691; doi:10.3390/molecules26164914)
Supplement: Supplementary file 1 [file molecules-26-04914-s001.zip › molecules-1292519-supplementary.pdf]

Supplementary Material

Morphological Changes and Component Characterization of Coffee Silverskin

Xia Wang [1,2,†], De-Fu Hong [1,2,†], Gui-Lin Hu [1,2,†], Zhong-Rong Li [1], Xing-Rong Peng [1], Qiang-Qiang Shi [1] and Ming-Hua Qiu [1,2,*]

[1] State Key Laboratory of Phytochemistry and Plant Resources in West China, Kunming Institute of Botany, Chinese Academy of Sciences, Kunming 650201, Yunnan, China; wangxia@mail.kib.ac.cn (X.W.); hongdefu@mail.kib.ac.cn (D.-F.H.); huguili@mail.kib.ac.cn (G.-L.H.); lizhongrong@mail.kib.ac.cn (Z.-R.L.); pengxingrong@mail.kib.ac.cn (X.-R.P.); shiqiangqiang@mail.kib.ac.cn (Q.-Q.S.)

[2] University of Chinese Academy of Sciences, Beijing 100049, China

* Correspondence: mhchiu@mail.kib.ac.cn; Tel.: +86-0871-6522-3327; Fax: +86-0871-6522-325

† These authors contributed equally to this work.

Table S1. NMR data acquisition conditions.

	Spectral width (Hz)	Number of scans	Empty sweep	Sampling points	Sampling data lattice
^{1}H-NMR	16025.64	16	2	65536	--
^{13}C-NMR	48076.92	2048	4	65536	--
^{1}H-^{13}C HSQC	8012.82	8	16	--	1024 × 400
^{1}H-^{13}C HMBC	8012.82	32	16	--	2048 × 400
^{1}H-^{1}H COSY	8012.82	8	16	--	2048 × 400
^{1}H-^{13}C HSQC-TOCSY	8012.82	32	16	---	2048 × 400

Table S2. Comparison between the compounds from unroasted (**B**) and roasted (**A**) coffee silverskin aqueous extracts.a.

Compound	Characteristic Peak (ppm)	Number of protons	Integral value		Relative content (%)		Rate of change (%)
**Sample**	–	–	**As**	**Bs**	**A**	**B**	**(As-Bs)/Bs×100%**
**Total integral value**	–	–	**11.90**	**21.90**	–	–	**– 45.6**
**TSP**	**0**	**9**	**1.00**	**1.00**	–	–	–
Carbohydrate	–	–	9.17	19.09	77.1	87.2	–52.0
Caffeine	7.85	1	1.05 ± 0.02	1.04 ± 0.04	8.8	4.7	+ 1.0
Trigonelline	4.42	3	3.46 ± 0.01	3.65 ± 0.03	9.7	5.6	– 5.2
5-chlorogenic acid	6.34	1	0.53 ± 0.03	0.55 ± 0.03	4.4	2.5	– 3.6
Sucrose	5.41	1	1.22 ± 0.03	2.63 ± 0.05	10.2	12.0	– 53.6
α-D-glucopyranose	5.22	1	0.94 ± 0.06	2.17 ± 0.02	7.9	9.9	– 56.7
β-D-glucopyranose	4.64	1	1.72 ± 0.06	3.43 ± 0.05	14.5	15.7	– 49.8
β-D-pyranose	3.99	1	4.13 ± 0.03	8.48 ± 0.12	34.8	38.7	– 51.3
β-D-fructofuranose	4.11	2	2.31 ± 0.09	4.76 ± 0.12	9.7	10.9	– 51.5

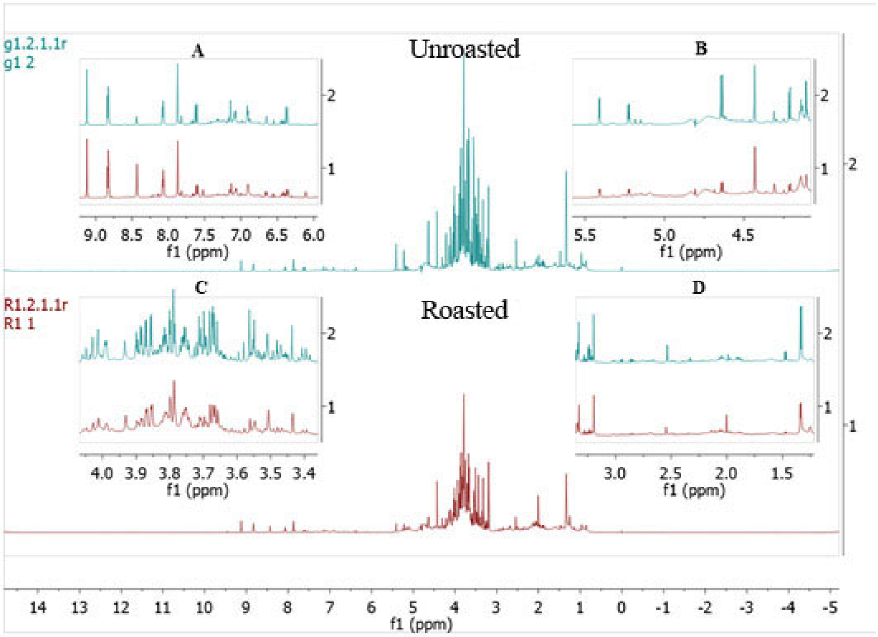

Figure S1. The comparison of ¹H NMR spectra between unroasted B1 (up) and lightly roasted A1 (down) CS aqueous extracts. Roasted and unroasted CS are similar in chemical composition, but the total content of unroasted CS is higher than roasted CS.

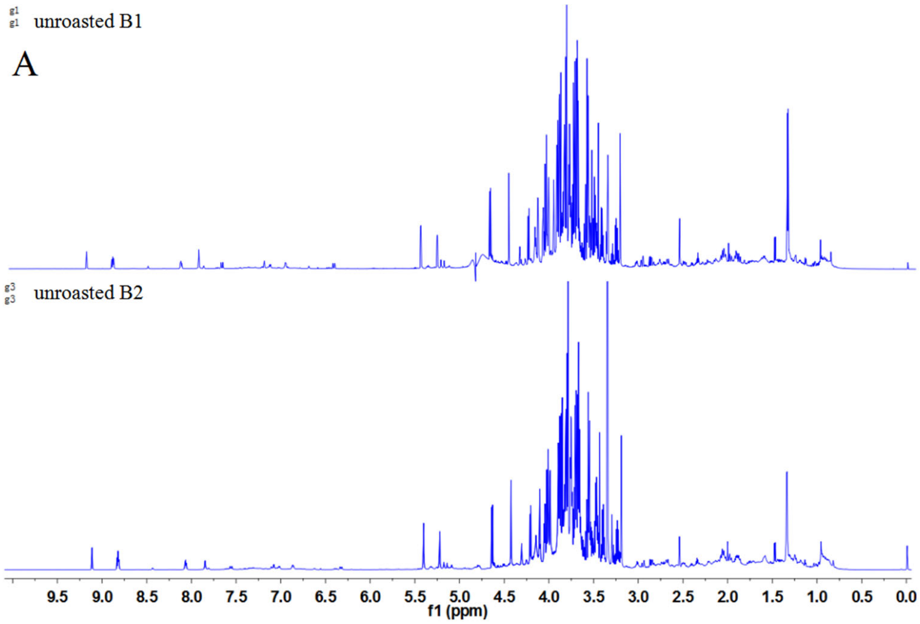

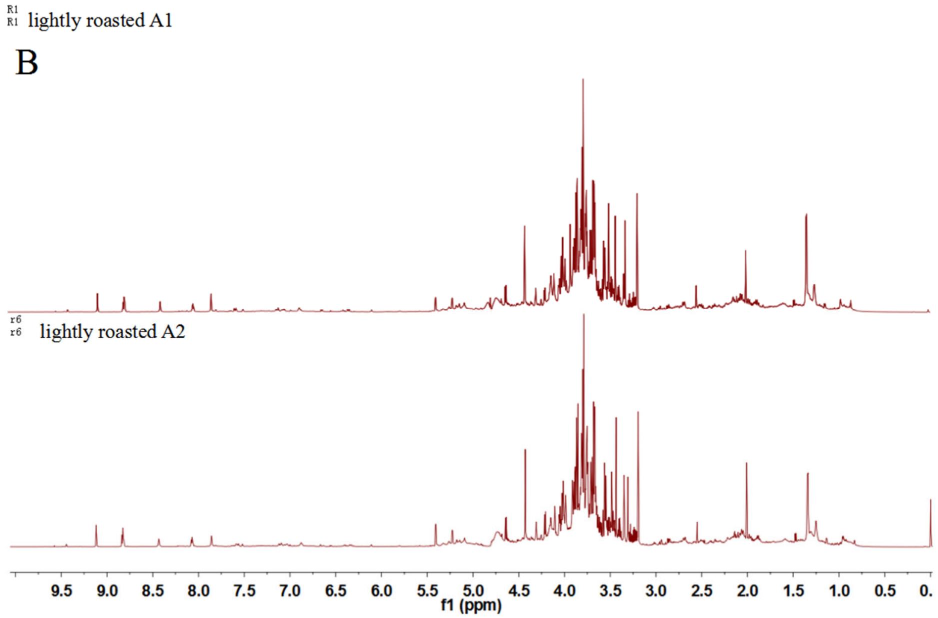

Figure S2. ^{1}H NMR spectrum signal of unroasted CS and lightly roasted aqueous extracts.

A

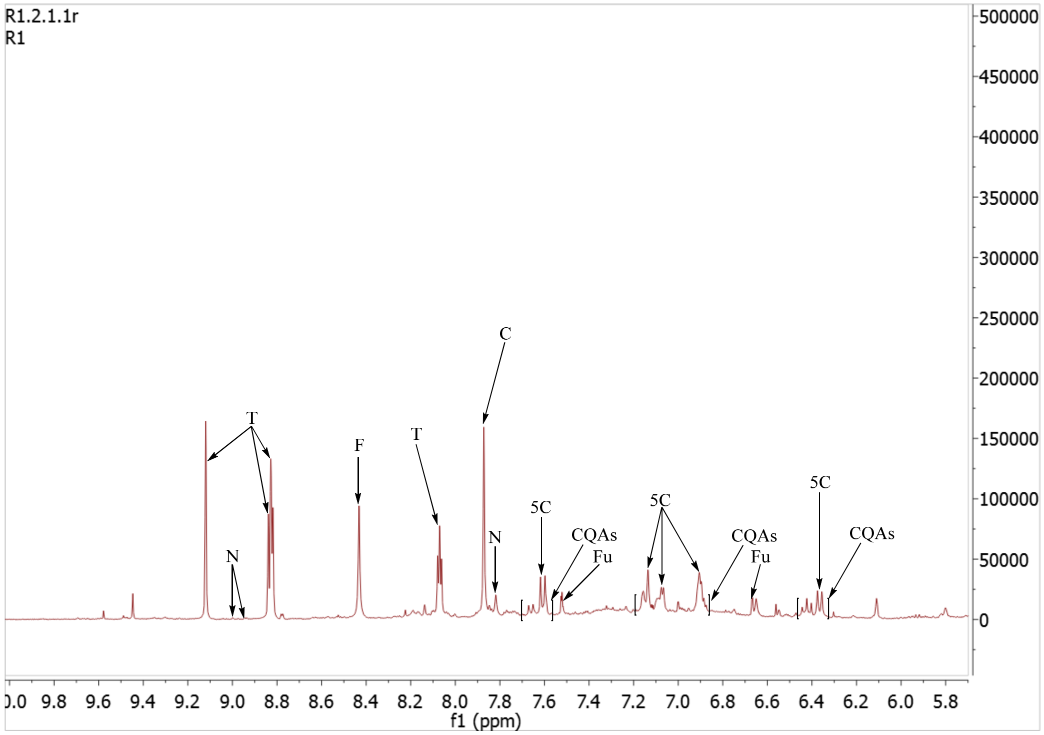
R1.2.1.1r
R1
T
C
F
T
N
5C
N
CQAs
Fu
5C
CQAs
Fu
5C
CQAs
f1 (ppm)

B

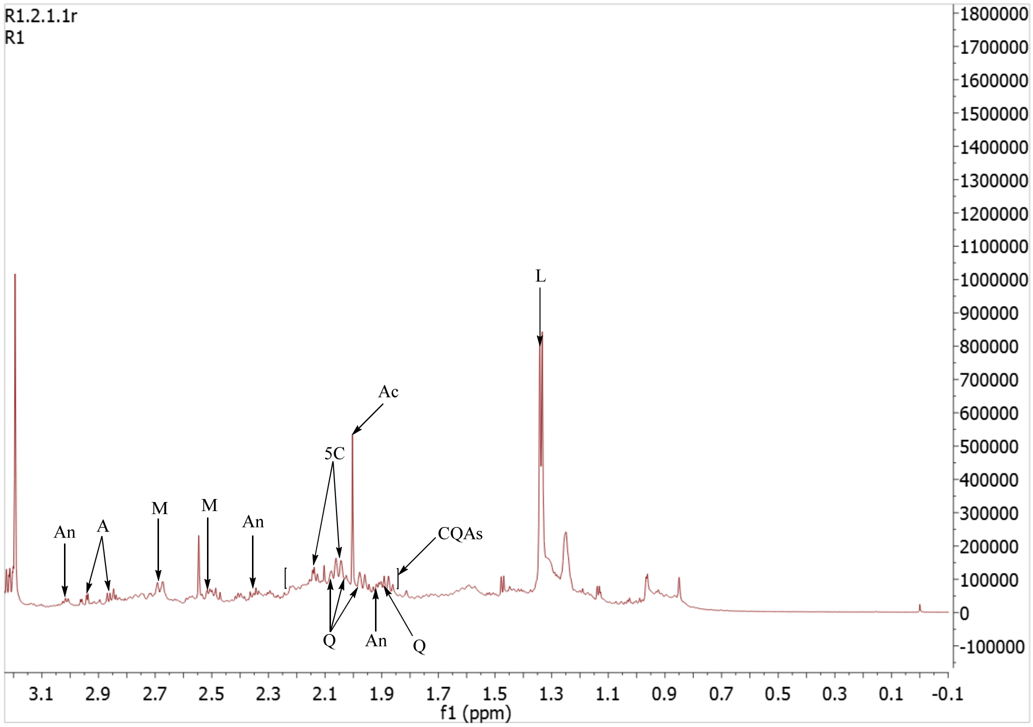
R1.2.1.1r
R1
L
Ac
5C
An
A
M
M
An
CQAs
Q
An
Q
f1 (ppm)

C

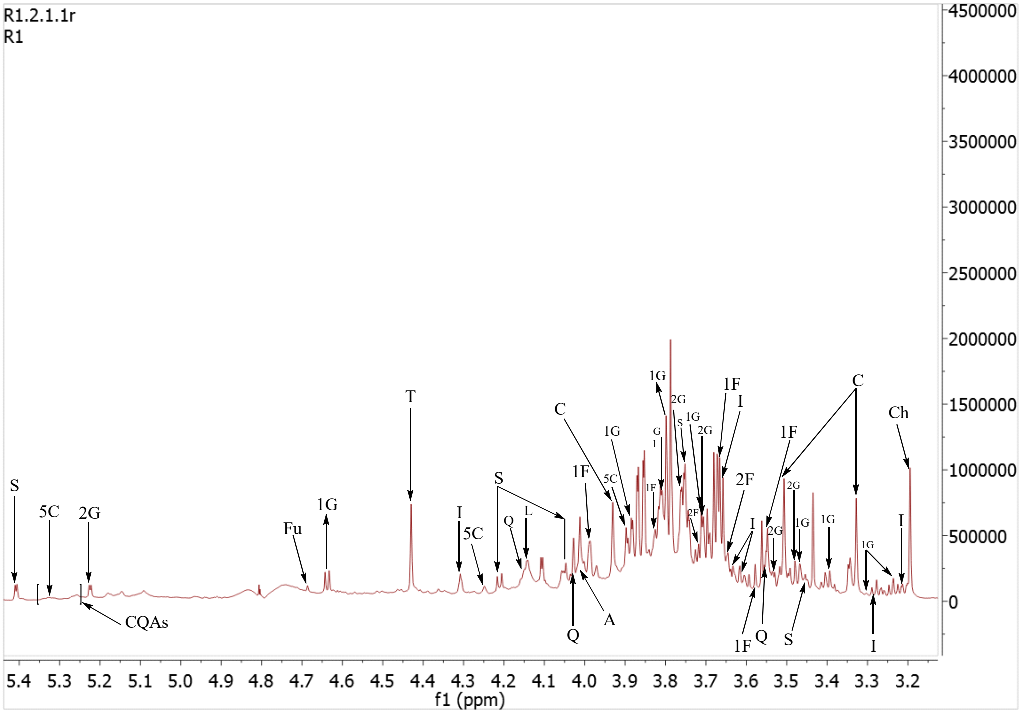

Figure S3. ^{1}H NMR spectrum signal identification of lightly roasted A1 CS aqueous extracts (A-C). caffeine (C)，trigonelline (T)，quinic acid (Q)，*β*-D-glucopyranose (1G)，*β*-D-fructofuranose (1F)，inostiol (I)，nicotinic acid (N)，asparagine (A)，choline (Ch)，glycine (Gl)，formic acid (F)，5-caffeoylquinic acid (5C)，*α*-D-glucopyranose (2G)，*β*-D-fructopyranose (2F)，sucrose (S)，2-furanylmethanol (Fu)，malic acid (M)，4-Amino-N-butyric acid (An)，lactic acid (L)，acetic acid (Ac).

A

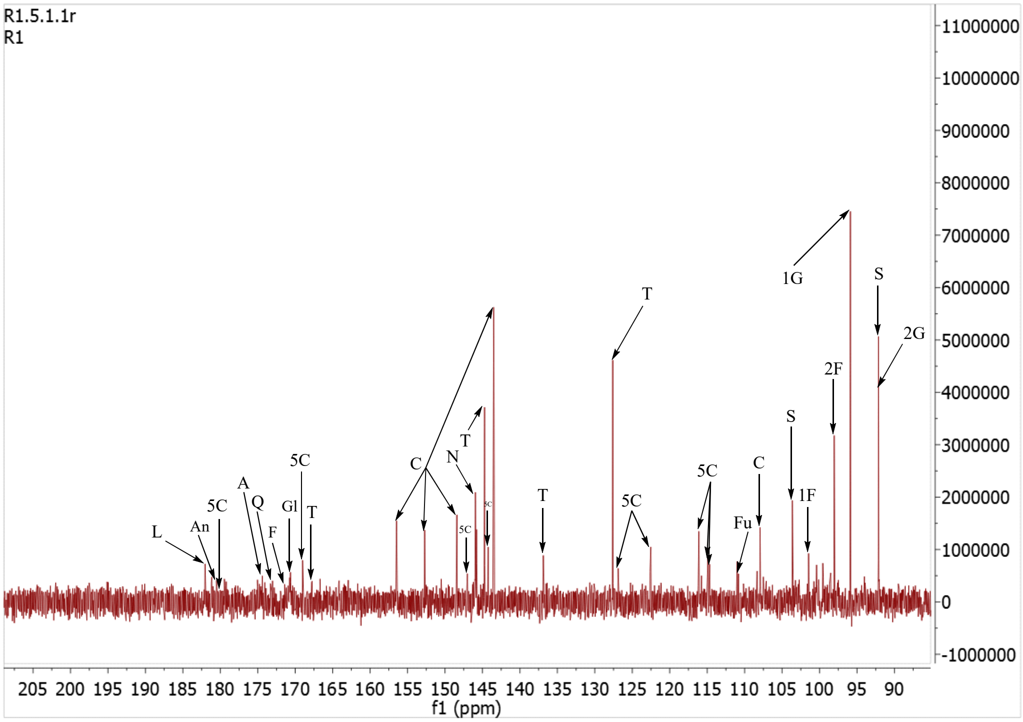

B

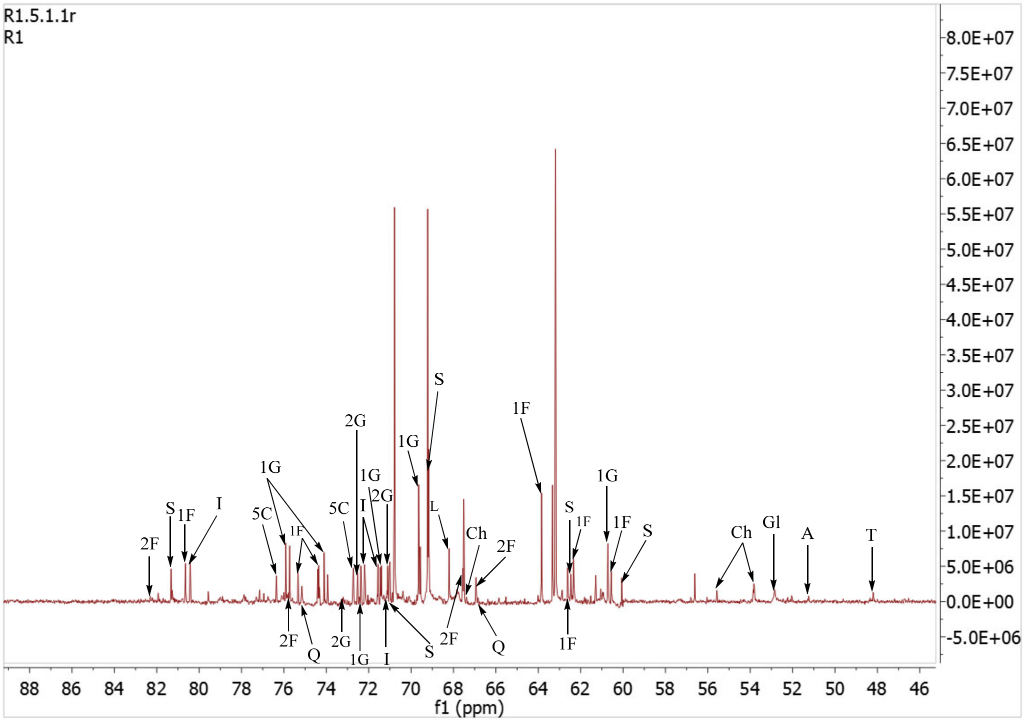

C

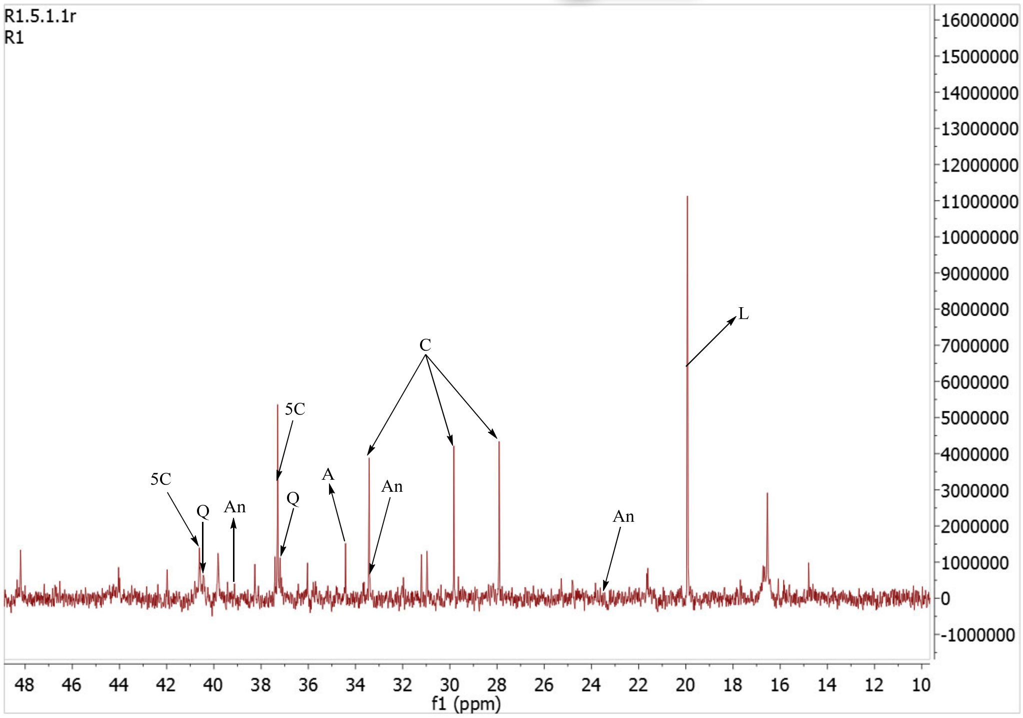

Figure S4. ^{13}C NMR spectrum signal identification of lightly roasted A1 CS aqueous extracts (A-C). caffeine (C), trigonelline (T), quinic acid (Q), *β*-D-glucopyranose (1G), *β*-D-fructofuranose (1F), inostiol (I), nicotinic acid (N), asparagine (A), choline (Ch), glycine (Gl), formic acid (F), 5-caffeoylquinic acid (5C), *α*-D-glucopyranose (2G), *β*-D-fructopyranose (2F), sucrose (S), 2-furanylmethanol (Fu), malic acid (M), 4-Amino-N-butyric acid (An), lactic acid (L), acetic acid (Ac).

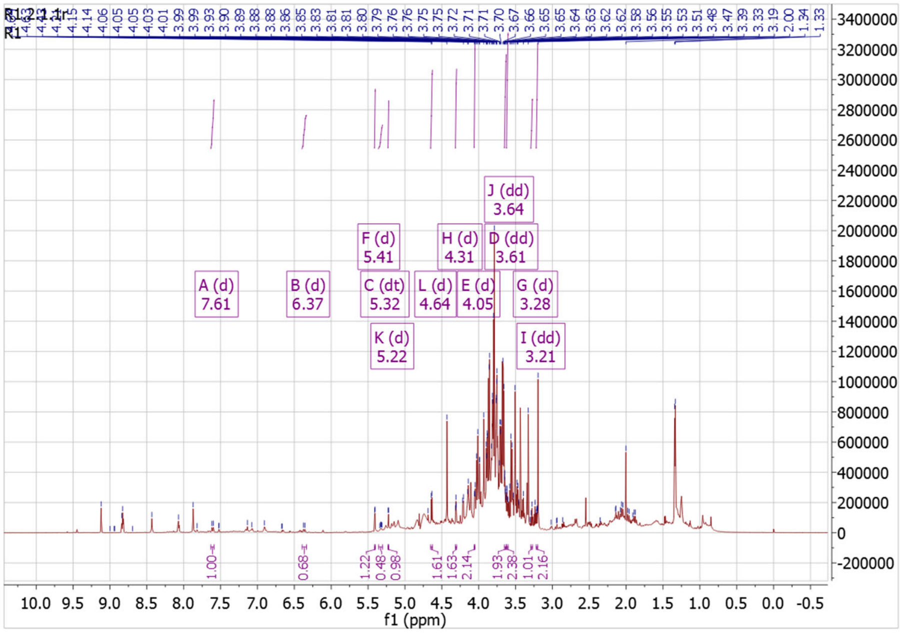

Figure S5. ^1H NMR spectrum of lightly roasted A1 CS aqueous extracts.

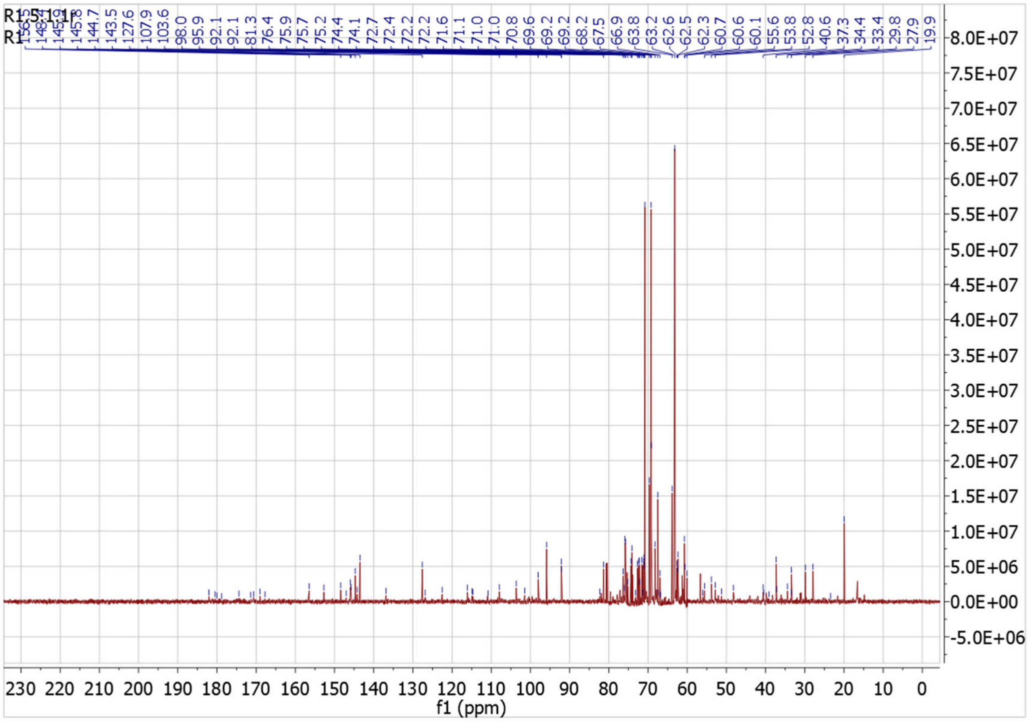

Figure S6. ^{13}C NMR spectrum of lightly roasted A1 CS aqueous extracts.

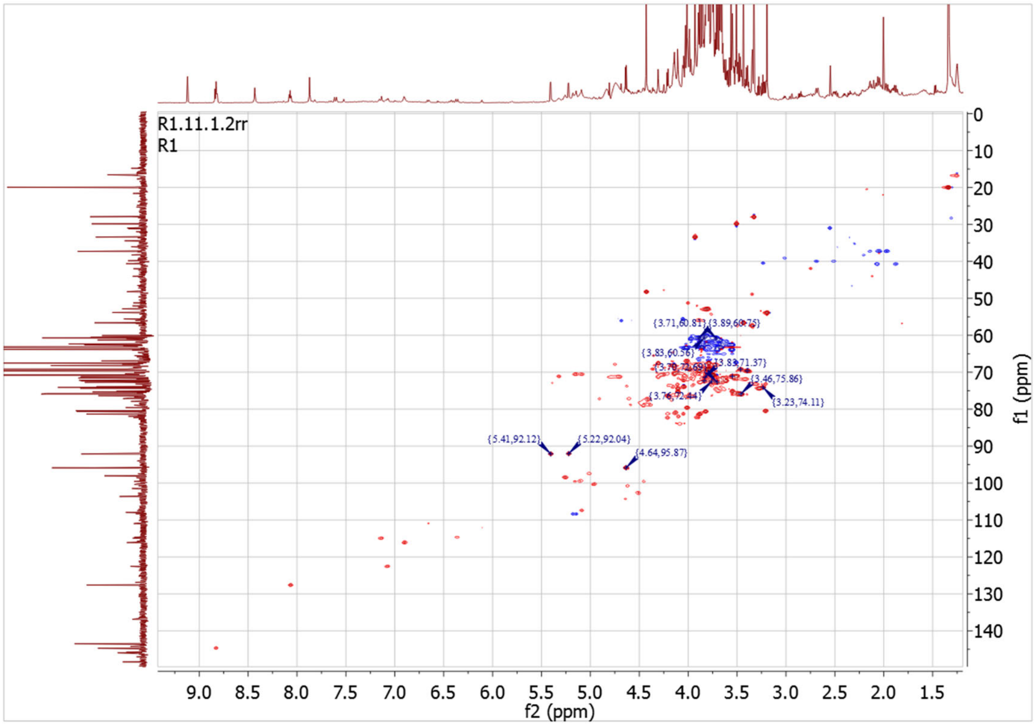

Figure S7. HSQC spectrum of lightly roasted A1 CS aqueous extracts.

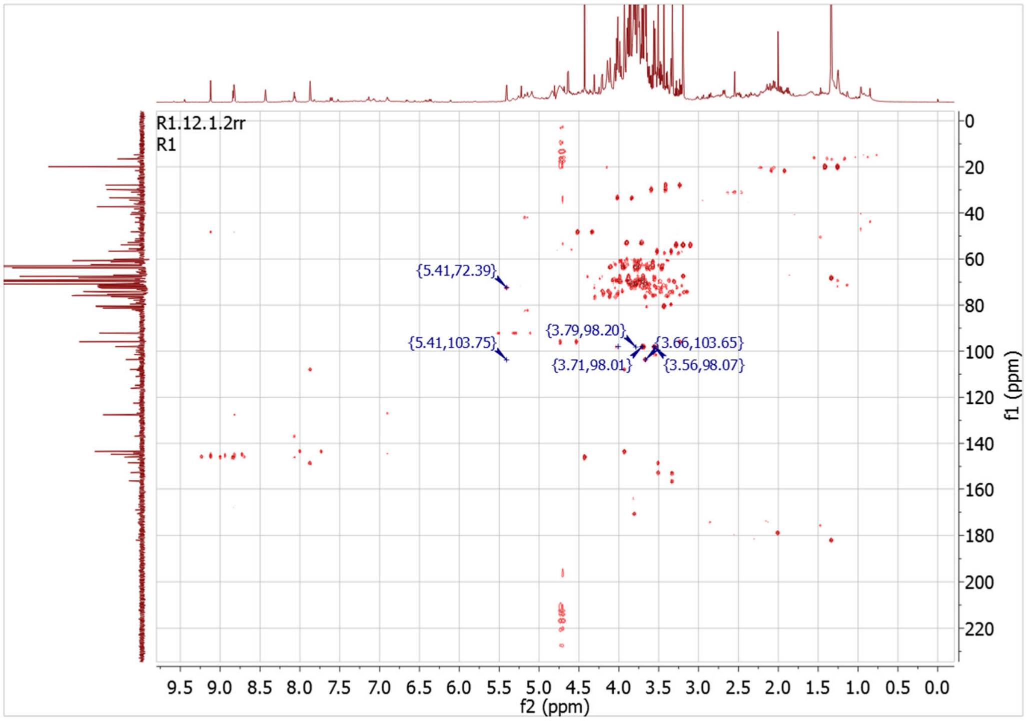

Figure S8. HMBC spectrum of lightly roasted A1 CS aqueous extracts.

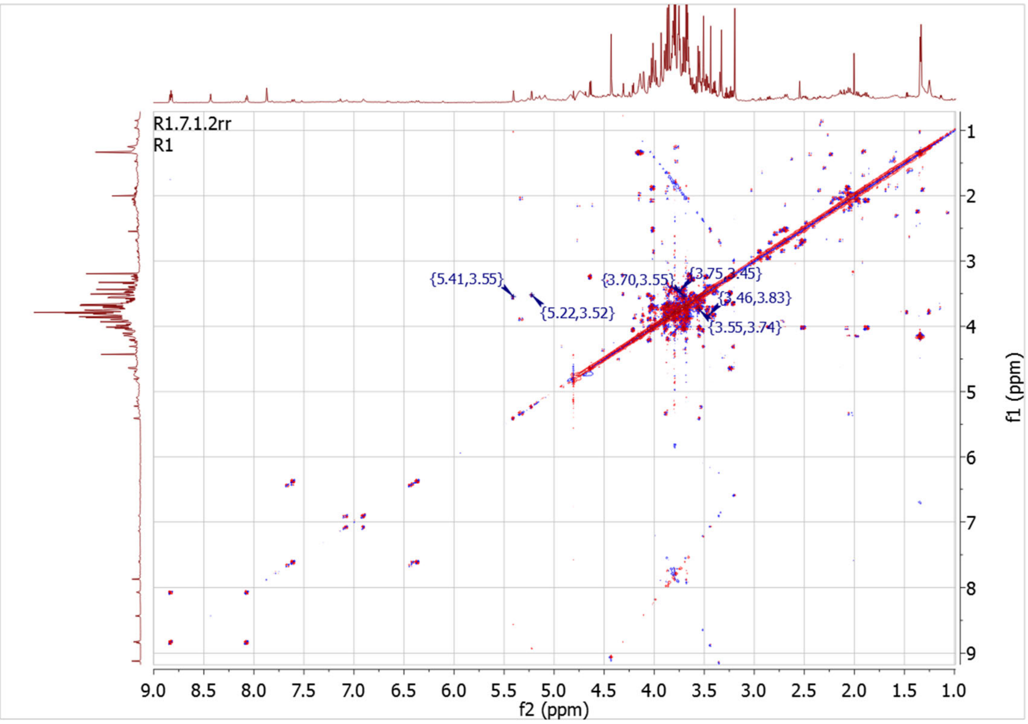

Figure S9. ^{1}H-^{1}H COSY spectrum of lightly roasted A1 CS aqueous extracts.

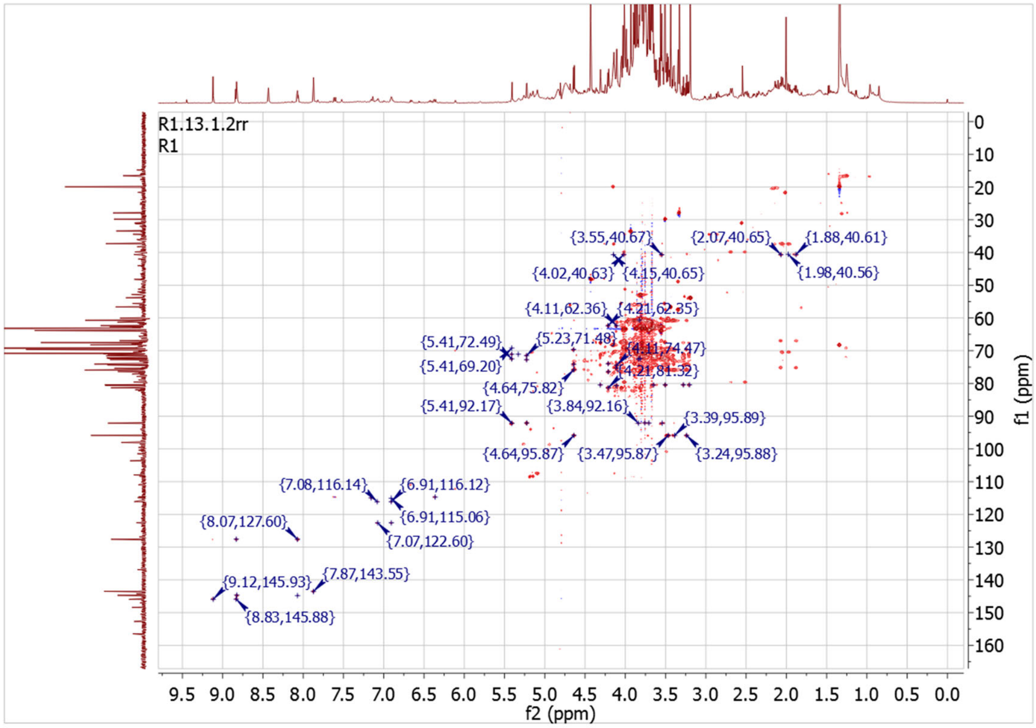

Figure S10. HSQC-TOCSY spectrum of lightly roasted A1 CS aqueous extracts.

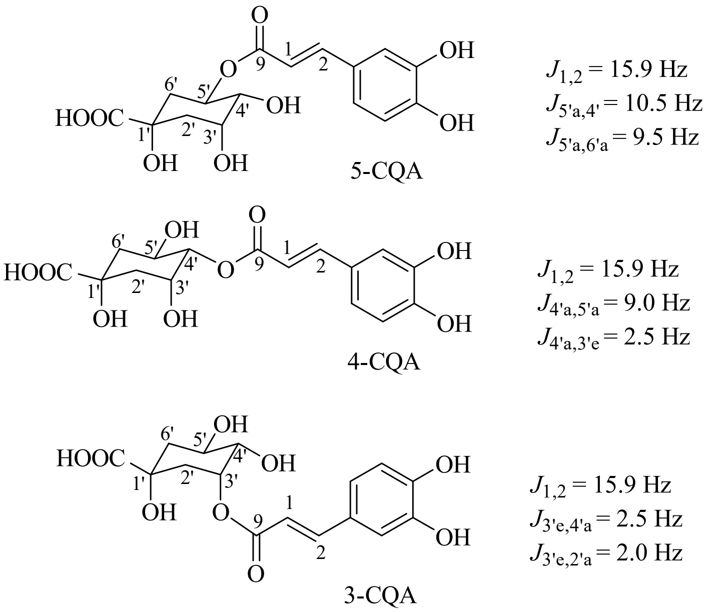

Figure S11. Characteristic coupling constants *J* for 3-CQA, 4-CQA, 5-CQA.

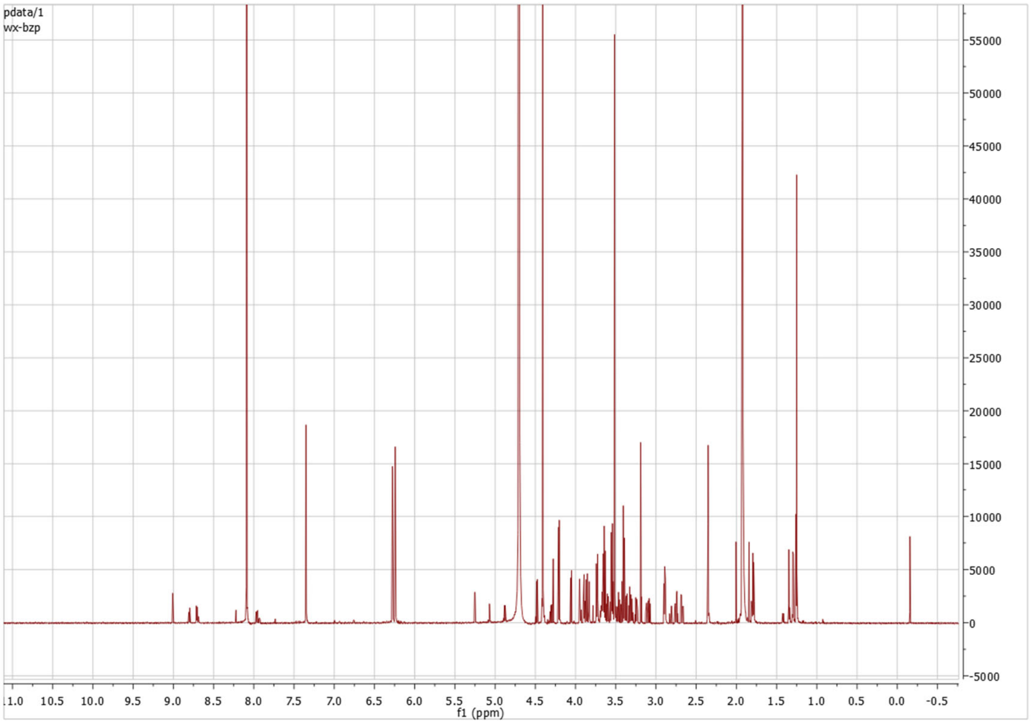

Figure S12. ^{1}H NMR spectrum of the mixed standards.

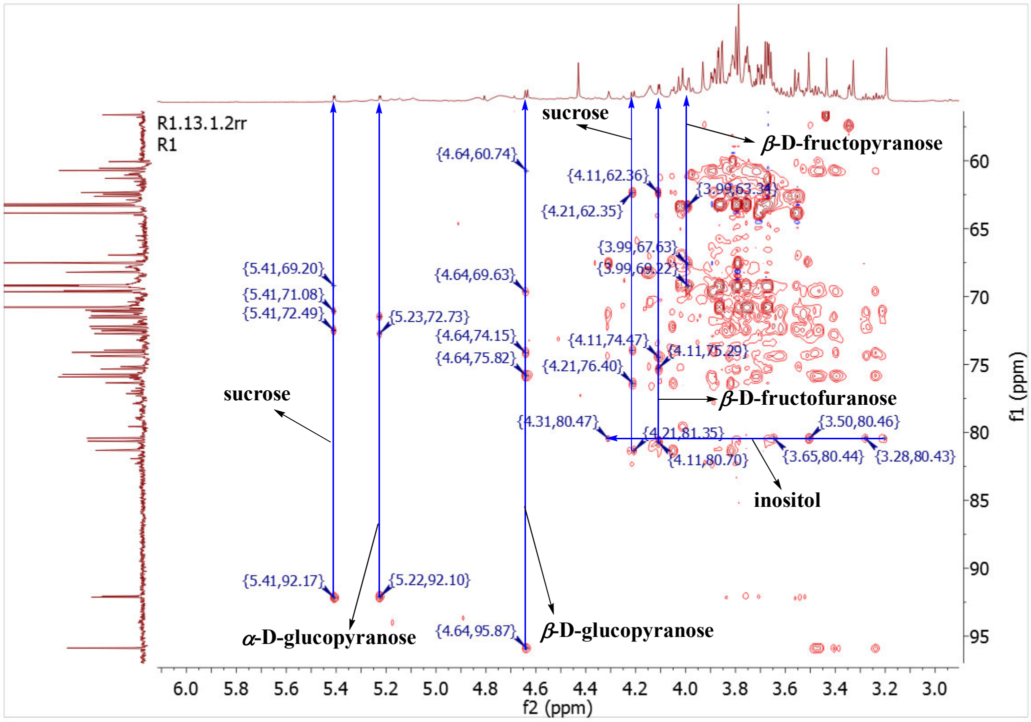

Figure S13. The HSQC-TOCSY correlations of carbohydrates from silverskin aqueous extract.

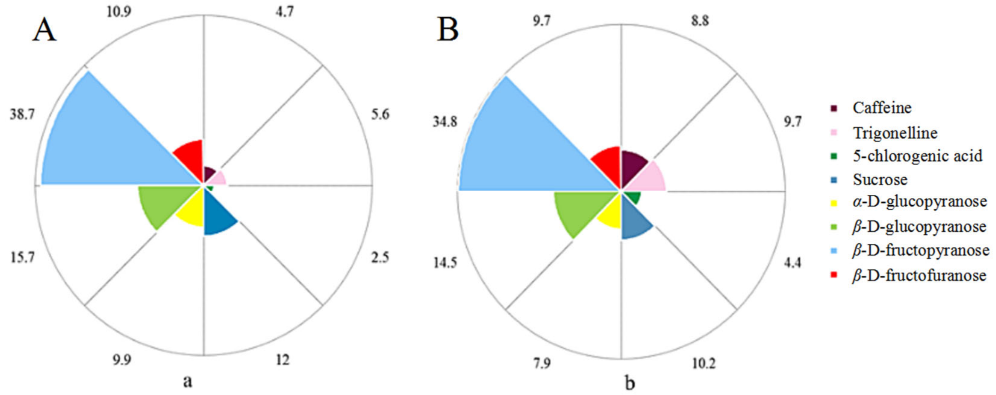

Figure S14. The relative content of different compounds before (A) and after roasting (B).
